# Immunotherapeutic Potential of m6A-Modifiers and MicroRNAs in Controlling Acute Myeloid Leukaemia

**DOI:** 10.3390/biomedicines9060690

**Published:** 2021-06-18

**Authors:** Sunil Kumar, Ravinder Nagpal, Amit Kumar, Muhammad Umer Ashraf, Yong-Soo Bae

**Affiliations:** 1 Department of Biological Sciences, Sungkyunkwan University, Jangan-gu, Suwon 16419, Gyeonggi-do, Korea; drumerashraf@gmail.com; 2 Science Research Center (SRC) for Immune Research on Non-lymphoid Organ (CIRNO), Sungkyunkwan University, Jangan-gu, Suwon 16419, Gyeonggi-do, Korea; 3 Department of Nutrition & Integrative Physiology, Florida State University, Tallahassee, FL 32306, USA; rnagpal@fsu.edu; 4 Medical Writer, Quebec City, QC G1X 3E1, Canada; amit.aiims2005@gmail.com

**Keywords:** epitranscriptomics, acute myeloid leukaemia, microRNA, CISH, immunotherapeutics

## Abstract

Epigenetic alterations have contributed greatly to human carcinogenesis. Conventional epigenetic studies have been predominantly focused on DNA methylation, histone modifications, and chromatin remodelling. Epitranscriptomics is an emerging field that encompasses the study of RNA modifications that do not affect the RNA sequence but affect functionality via a series of RNA binding proteins called writer, reader and eraser. Several kinds of epi-RNA modifications are known, such as 6-methyladenosine (m6A), 5-methylcytidine (m5C), and 1-methyladenosine. M6A modification is the most studied and has large therapeutic implications. In this review, we have summarised the therapeutic potential of m6A-modifiers in controlling haematological disorders, especially acute myeloid leukaemia (AML). AML is a type of blood cancer affecting specific subsets of blood-forming hematopoietic stem/progenitor cells (HSPCs), which proliferate rapidly and acquire self-renewal capacities with impaired terminal cell-differentiation and apoptosis leading to abnormal accumulation of white blood cells, and thus, an alternative therapeutic approach is required urgently. Here, we have described how RNA m6A-modification machineries EEE (Editor/writer: Mettl3, Mettl14; Eraser/remover: FTO, ALKBH5, and Effector/reader: YTHDF-1/2) could be reformed into potential druggable candidates or as RNA-modifying drugs (RMD) to treat leukaemia. Moreover, we have shed light on the role of microRNAs and suppressors of cytokine signalling (SOCS/CISH) in increasing anti-tumour immunity towards leukaemia. We anticipate, our investigation will provide fundamental knowledge in nurturing the potential of RNA modifiers in discovering novel therapeutics or immunotherapeutic procedures.

## 1. Introduction

There are several conventional drugs, including epigenetic-based drugs targeting DNA and histone modifications, that have been already approved by the US food and drug administration (FDA) for the treatment of acute myeloid leukaemia (AML). Targeting RNA-modification machineries as RNA-modifying drugs (RMDs) refuelled the scientific interest to discover novel therapeutic drugs. Although RNA modification was discovered as early in the 1970s [1,2], it refuelled the passion of scientific research after the discovery of the first m6A-demethylase in 2008 [3]. To date, more than 150–170 RNA chemical modifications have been identified [4,5,6] in methylating approximately 7000 human transcripts; among them, 50% is conserved in mice [7]. The majority of m6A-modification takes place in the coding region of mRNA enriching near 5`UTR and 3`UTR requiring conserved DRACH (D = G/A/U; R = G/A; H = A/C/U) sequences [8,9,10]. The three major RNA epigenetic modifiers reforming the whole biological functions are writers, erasers, and readers (Box 1). The detailed mechanism of m6A-modification and gene regulation is well-described in several excellent review articles [4,6,7]. However, this review mainly focuses on reforming RNA epigenetic machineries into druggable form to treat haematological disorders especially AML [11].

## 2. AML Therapeutics

**Figure 1:**
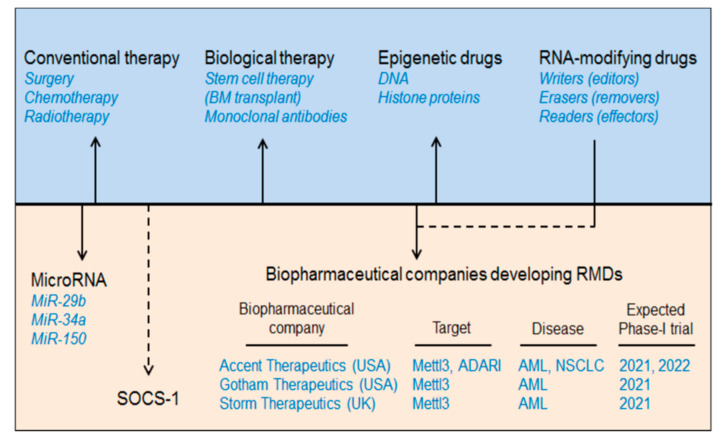
Development of AML therapeutics. The major AML therapeutics are depicted in the diagram above which includes conventional therapy (chemotherapy, radiotherapy, and surgery), biological therapy (stem cell therapy or bone marrow transplant), epigenetic drugs (DNA and histone modifier-based drugs), RNA-modifying drugs (writers/editors, erasers/removers, and readers/effectors), microRNAs (miR-29b, miR-34a, and miR-150) and suppressor of cytokine signalling (SOCS1/CISH). Additionally, we have mentioned some biopharmaceutical companies developing RMDs and estimated phase-I clinical trials. ATRA: all-trans retinoic acid.

AML is a group of neoplastic diseases involving bone marrow with or without the involvement of peripheral blood. It is the most common type of haematological disorder prevalent in children and adults, globally affecting nearly three million people every year [11]. For some time now, several conventional therapies have been employed to treat AML including chemotherapy and, rarely, surgery and radiation [11]. Cytotoxic chemotherapy or remission-induction with chemotherapeutic agents (anthracycline and cytarabine) followed by consolidation therapy involving an allogeneic stem cell transfer, bone marrow transplant (BMT), or hematopoietic stem cell transplantation have been used as a standard therapy regimen to control remission of AML [12]. However, due to the delayed diagnosis of AML and the therapy-related morbidity and mortality, these conventional means of treating AML have fallen short when it comes to a “do no harm” approach. Recently, with the advancement of next-generation sequencing and gene-mutational analysis, several new strategies have been under development to minimise the global harmful effects of these conventional therapies [13,14]. One of these new strategies includes the ‘targeted-drug therapy” which is cell-, gene-, or marker-specific and allows the option of treatment in patients where invasive chemotherapy is not feasible. This targeted therapy includes biological (monoclonal antibodies), epigenetic or combination therapies [15] (Figure 1, Table 1). Several antigen-specific monoclonal antibodies have now been approved by the FDA for AML therapy. Among these, CD33-directed Gemtuzumab ozogamicin (GO) mAb was the first to be used for AML therapy [16]. In addition, several studies have now emerged marking DNA methylation as one of the hallmarks of AML carcinogenesis [17] involving DNA methyltransferases (DNMTs) and Ten-eleven-translocation (TET) dioxygenases [18]. Various DNMT inhibitors have been investigated for AML therapy. However, only two of these (azacitidine and decitabine) are FDA approved [19]. Any detrimental mutation in the TET family members results in altered DNA methylation [20]. For example, isocitrate dehydrogenases (IDH1 and IDH2) can inhibit TET2 and can cause hypermethylation resulting in AML progression [21]. Therefore, IDH inhibition can resolve this TET2 reduction-induced hypermethylation [22]. The FDA has approved two oral IDH inhibitors: ivosidenib (AG-120) and enasidenib (AG-221) for AML treatment [23,24]. In addition to DNA modifiers, histone modifiers (HATs and HDACs) have also been implicated in AML treatment [25] by regulating tumour suppressor genes [26,27]. At present, four HDAC inhibitors (HDACi) have been approved by the US FDA including three as panHDACi: belinostat, vorinostat, panobinostat, and romidepsin as a selective HDACi [28]. We have summarised the current biopharmaceutical companies developing AML therapeutics as RNA modification drugs (RMDs) in Figure 1.

## 3. Epitranscriptomics in AML Therapy

This section describes the strategies to treat haematological disorders, especially AML by targeting intracellular RNA-modification machineries, like EEE (Editor/writer: Mettl3, Mettl14; Eraser/remover: FTO, ALKBH5 and Effector/reader: YTHDF-1, YTHDF-2) (Box 1), microRNAs and Suppressor of Cytokine Signalling family of proteins (SOCS/CISH) and describing how these can be developed as personalized medicines (Figure 1).

### 3.1. Editors (Writers)

#### 3.1.1. Mettl3 in AML

Vu et al., 2017 have demonstrated the role of m6A writer enzyme ‘Mettl3’ in AML progression [43] supported by Barbieri et al., 2017 [44] and enlightened the molecular mechanism to control leukaemia. The authors found that ‘Mettl3′ is normally expressed in CD34+ hematopoietic stem/progenitor cells (HSPCs) but aberrantly expressed in leukaemia cells as compared to the other tumour-types. However, Mettl3-silencing in HSPCs promoted HSPCs differentiation and apoptosis but inhibited self-renewal capacity and proliferation. Conversely, Mettl3 overexpression inhibited cell-differentiation and apoptosis but allowed cell-proliferation and self-renewal capacities [43]. These results suggest that Mettl3 facilitates AML cell growth but resist cell-differentiation. Furthermore, it was also reported that the adoptive transfer of Mettl3-deficient human AML cell line (MOLM-13) into immunodeficient (recipient) mice induces cell-differentiation and apoptosis but remarkably delayed leukaemia progression. This result further supports the key role of Mettl3 in AML progression. Mechanistically, authors justified (via m6A-mapping and ribosome profiling) that Mettl3 increases the expression of c-Myc, Bcl2 (apoptosis regulator) and pTEN (a proto-oncogene or AKT regulator) genes by m6A-modification in human leukemic MOLM-1 cell-line. Moreover, Mettl3 regulates AKT-RICTOR signalling (PI3K-AKT) pathway and contributes to AML progression by increasing cell-proliferation and decreasing cell-differentiation capacities. The loss of Mettl3 activates pTEN-mediated AKT phosphorylation and controls AML by reducing cell-proliferation and promoting cell-differentiation. Vu et al., 2017 demonstrated the Mettl3-deficient AML cells treated with PI3K inhibitor showed reduced cell-differentiation [43]. Taken together, these investigations confirm the key role of Mettl3 in facilitating AML growth and survival and potentiate the therapeutic value of Mettl3 in controlling AML by means of either specific inhibition or selective intracellular silencing in AML. These findings were further supported by Konstantinos et al., 2019 [45]; by synthesizing two Mettl3-inhibitors that inhibits AML cell expansion (Table 2). Moreover, targeting AKT-RICTOR signalling pathways by PI3K-inhibitors in combination with other forms of conventional (radio/chemo/stem cell transplant) treatments could overcome AML progression [45,46,47].

#### 3.1.2. METTL14 in AML

**Figure 2:**
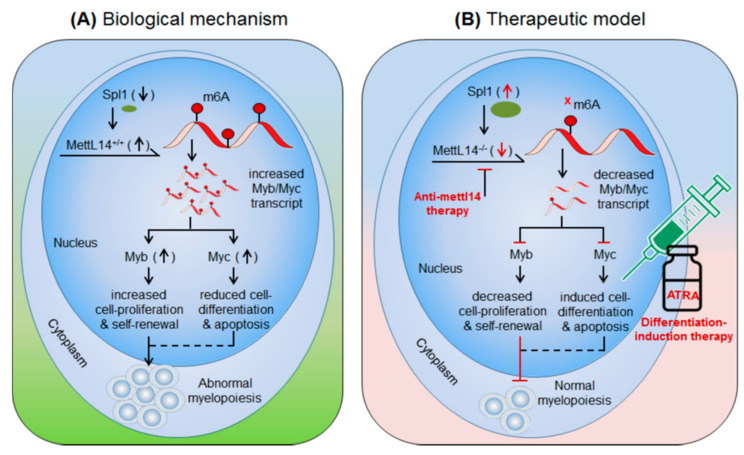
Therapeutic model targeting intracellular m6A-writer enzyme “Mettl14” in controlling AML. (**A**) Biological mechanism: Mettl14 methylates and enhances the expression of Myb and Myc transcription factor. The elevated MYB/MYC increases cell-proliferation and self-renewal capacity and reduces cell-differentiation and apoptotic processes, resulting in abnormal myelopoiesis. (**B**) Therapeutic model: Anti-Mettl14 therapy: intracellular silencing of Mettl14 decreases the expression of Myb/Myc TcFs resulting in decreased cell-proliferation and improved cell-differentiation and apoptotic processes. Differentiation-induction therapy: all-trans retinoic acid (ATRA) further enhances cell-differentiation and apoptotic processes and thus allows normal myelopoiesis. Targeting other pathways like; Spl1, Myb/Myc, AKT pathways have been also proposed previously [62,63] in controlling AML.

Weng et al., 2018 [49] demonstrated the role of m6A-writer enzyme ‘Mettl14′ in AML progression. Mettl14′ is aberrantly expressed in AML patients (data from Cancer Genome Atlas (TCGA): portal.gdc.cancer.gov/) and several leukaemia cell lines and AML cells carrying *t*(11q23), *t*(15;17), or *t*(8;21) translocation)-mutations [49]. Of note, Mettl14 is downregulated during myeloid differentiation [49]. These findings suggest the prominent role of Mettl14-mediated m6A-modifications in leukaemia progression. In-vivo depletion of Mettl14 in recipient conditional knockout mice Mettl14^cKO^ or Mettl14^fl/fl^-CRE^ERT^ mice decreases cell proliferation and self-renewal capacity of CD45.2^+^ (of Mettl14^cKO^) in peripheral blood as compared to the CD45.1^+^ CD45.2^+^ (competitor) cells [49]. However, in-vitro depletion of Mettl14 in human HSPCs/-CD34^+^ promotes cell-differentiation demonstrated by increase in differentiation markers (CD11b in monocytes and CSF1R in macrophages). These findings suggest that Mettl14 induces leukaemia growth by increasing cell-proliferation and decreasing terminal cell-differentiation. Conversely, the differentiation-inducing agent (OP9 culture medium) [55], PMA [56] and all-trans retinoic acid (ATRA) [57] significantly decrease the expression of Mettl14 and overall m6A-abundance in AML cells. Reciprocally, Mettl14 overexpression inhibits myeloid differentiation. Mettl14 affects the mRNA stability of several transcription factors that play important role in the progression of several types of cancers. Weng et al., 2018 showed that Mettl4 increase the stability of *Myb* and *Myc* transcripts [49] and SP1 negatively regulates the expression of Mettl14 [49]. Previously several independent reports confirmed the high levels of MYB and MYC in leukaemia and lymphomas [58,59,60,61]. These findings suggest an important role of Mettl14 in AML progression by decreasing cell-differentiation and increasing cell-proliferation. Furthermore, SP1-Mettl14-MYB/MYC axis plays an important role in AML pathogenesis [49]. Taken together, above findings suggests Mettl14 in combination with various differentiation-inducing agents such as ATRA could be a promising approach to control AML with the other forms of therapeutic approaches (radio/chemo/BM-transplantation) [49], (Figure 2, Table 2).

### 3.2. Erasers (Removers)

#### 3.2.1. m6A-Demethylase FTO in AML

**Figure 3:**
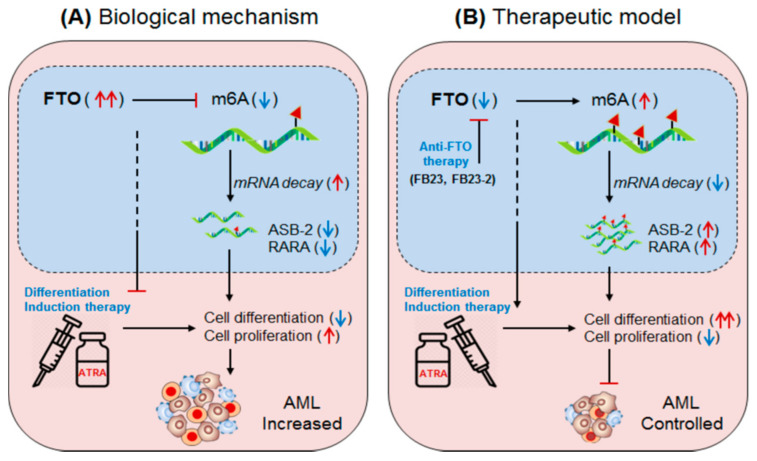
Therapeutic model targeting intracellular m6A-eraser protein FTO in controlling AML. (**A**) Biological mechanism: The m6A-demethylase FTO is abnormally expressed in APL and negatively regulates the expression of its target genes (ASB2 and RARA) causing decreased ATRA-induced cell-differentiation and increased self-renewal capacity of acute promyelocytic leukaemia (APL) cells, resulting in rapid APL progression. (**B**) Therapeutic model: Anti-FTO therapy: targeting FTO by means of either selective inhibitor (FB23 and FB23-2) [75] or intracellular silencing (shFTO) efficiently control APL by inducing m6A-mediated increase of ASB2 and RARA genes, as well as by enhancing the efficacy of ATRA-mediated differentiation and apoptosis [69].

Li, et al., 2017 [51]; have demonstrated the oncogenic role of FTO, an m6A-demethylase, in facilitating leukemogenesis by promoting cell-proliferation (self-renewal) and reducing cell-differentiation and apoptotic processes. FTO is aberrantly expressed in a specific subset of hematopoietic stem/progenitor cells (HSPCs/CD34^+^ CD38^−^) carrying *t*(11q23)/MLL-rearrangement and *t*(15;17)/PML-RARA mutations, called acute promyelocytic leukaemia (APL) [51]. FTO affects m6A methylation levels of several transcripts involved in leukemogenesis and cellular transformation. Several independent studies suggest that FTO regulates the expression of ankyrin repeat and SOCS box protein 2 (ASB2) [50,51], and retinoic acid receptor alfa (RARA) [64]. These targets of FTO have been confirmed by several experimental approaches [65,66,67,68]. Collectively these findings indicate that FTO regulate its target gene expression by its m6A-demethylase activity. However, under hypo-methylated condition FTO controls its targets by modulating their target’s transcripts stability. For instance, FTO overexpressing cell line showed increased mRNA-decay, suggesting the mechanism of target gene control by destabilizing the mRNA stabilities. Furthermore, under diseased state, the aberrantly expressed FTO negatively regulate its target gene expression; neither by directly decreasing m6A-level (hypo-methylation) of the target mRNA nor by m6A-effector protein YTHDF2-mRNA decay-dependent mechanisms, but by directly affecting the mRNA stability via its demethylase activity [58]. However, the possibility of m6A-reader protein (YTHDF1 and YTHDF2)-mediated regulation of ASB2 and RARA also cannot be completely denied. This is due to the fact that under YTHDF1 knockdown condition the expression of target genes (ASB2 and RARA) increases. Nevertheless, the FTO-mediated APL progression is not only by targeting mRNA destabilization, but also by affecting the efficacy of ‘ATRA-mediated therapy’ [69,70] justified by analyzing NB4 cell (human acute promyelocytic leukaemia cell line) differentiation marker. The overexpression of FTO significantly inhibits ATRA-induced cell-differentiation of NB4 cells [71] characterized by decreased (CD11b^+^ CD14^+^) and increased (CD11b^−^ CD14^−^) markers. Conversely, the induction of ATRA significantly decreased FTO and thereby rescued/increased the expression of its target ASB2 and RARA genes [72,73]. Moreover, forced expression of ASB2 and RARA also increases cell-differentiation. Collectively, these results authenticate that FTO plays a crucial role in promoting AML (i) by inhibiting the efficacy of ATRA-mediated induction of cell-differentiation and apoptosis, and (ii) by suppressing its target gene expression. Therefore, targeting FTO could be a recommendable approach to control APL with other forms of chemotherapeutic drug combinations [51,74] (Figure 3, Table 2 and Table 3).

#### 3.2.2. ALKBH5 (Eraser) in AML

Shen et al., 2020 [52] have demonstrated the role of the other m6A-eraser protein α-ketoglutarate-dependent dioxygenase AlkB homolog 5′ (ALKBH5) in facilitating leukaemia stem/initiating cells (LSCs/LICs) progression, a subset of AML characterized by high self-renewal capacity. Like the other m6A-eraser protein FTO [51], high expression of ALKBH5 has been reported in various subtypes of AML cells carrying *t*(15;17), Inv(16), *t*(8;21) and *t*(11q23) mutations, independent of specific TP53-mutation (in MONOMAC-6/MMC6, NOMO1, and NB4) and TP53-wild type (in MA9.3-ITD and MOLM13) human cell lines [52]. However, in vitro silencing of ALKBH5 in NOMO1 and MMC6 human cell lines significantly promotes apoptosis and restricts cell growth/proliferation. Furthermore, ex-vivo conditional knockout of ALKBH5 in MOLM13 cells also promoted apoptosis and reduced cell growth/proliferation. Conversely, overexpression of wild-type ALKBH5 reversed this effect and promoted AML-cell progression as compared to the mutant (H204A) ALKBH5, suggesting the crucial role of ALKBH5 in facilitating leukemogenesis. In addition, this also significantly increases the abundance of global m6A-level, as observed in the bone marrow of ALKBH5-depleted mice compared to the wild-type mice. Moreover, marked increase in global m6A-level was also noticed in both in vitro/ex vivo ALKBH5-depleted cell lines. These results suggest that (i) the m6A-demethylase ALKBH5 facilitate AML progression and required for its growth and survival. (ii) ALKBH5-depletion enhances global m6A-level.

To validate the requirement of ALKBH5 in the promotion/transformation of AML cells, Krivtsov et al., 2006 used MLL-AF9 (MA9)-induced leukemogenesis model (where MLL-rearranged fusion protein alone is sufficient to transform normal HSPCs into leukemic cells coupled with ALKBH5 knockout (ALKBH5^KO^) model [91]. ALKBH5-depletion significantly inhibited MA9-mediated cell immortalization [91]. On the contrary, forced expression of ALKBH5 significantly promoted MA9-mediated cell immortalization compared to the mutant ALKBH5 confirmed by in vitro colony formation/immortalization assays. The adoptive transfer (or BMT) of ALHBH5-deficient immune population (donor: CD45.2^+^Lin^−^) co-cultured with MLL-AF9 cells into recipient (CD45.1) mice significantly delayed leukaemia progression and prolonged survival with decreased splenomegaly, white blood cells count and immature blast cell (CD11b (Mac-1)^+^ c-Kit^+^) populations.

Moreover, ALKBH5-deletion inhibited the engraftment of MA9-transformed donor cells in the peripheral blood as compared to the ALHBH5-wild type donor under lethal irradiation conditions. These investigations suggest that ‘ALKBH5’ plays a key role in promoting leukemogenesis of a specific subset of AML cells. The relevant experiments on leukaemia xenograft mouse model further potentiate the involvement of ALKBH5 in self-renewal and maintenance of AML-cells.

**Figure 4:**
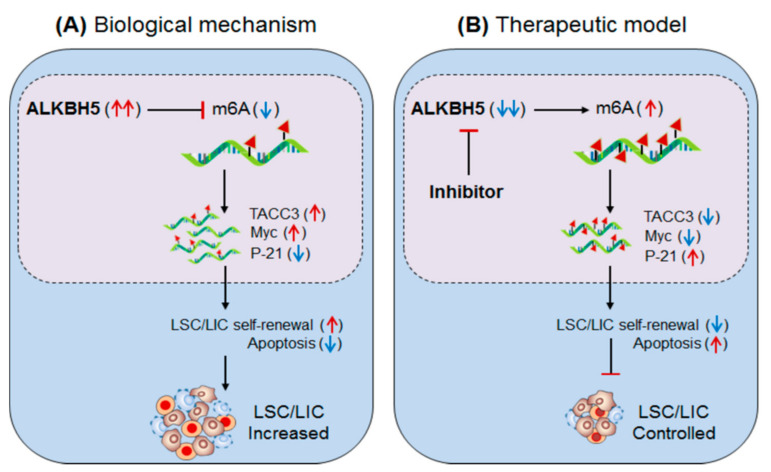
Therapeutic model targeting intracellular m6A-eraser protein ‘ALKBH5′ in controlling AML). (**A**) Biological mechanism: The m6A-demethylase ‘ALKBH5′ is aberrantly expressed in AML, especially in leukaemia stem/initiating cells (LSC/LIC) and facilitates its progression, indicating its requirement for development, self-renewal, maintenance and propagation. Biologically, the enhanced ALKBH5 in disease-state increases the expression of *TACC3* gene by m6A-mediated mechanism, resulting in increased self-renewal capacity and thereby increased progression of LSC/LICs. (**B**) Therapeutic model: Anti-ALKBH5 therapy: The selective inhibition of ALKBH5 decreases m6A methylation-mediated expression of TACC3 gene resulting in decreased self-renewal and increased apoptosis is well-efficient to control AML subsets.

RNA sequencing of ALKBH5-depleted MOLM13 and NOMO1 cells revealed TACC3 (transforming acidic coiled-coil-containing protein3) could be a positive target of ALKBH5 [52]. Furthermore, m6A-sequencing revealed enrichment of m6A-abundance at TACC3 mRNA in ALKBH5-depleted AML cells. ALKBH5 silencing decreases the expression of TACC gene, due to hyper-methylation of m6A, confirmed by decreased half-life of TACC3 (in MOLM13: 2.35 h–1.56 h and NOMO1: 3.4 h–1.55 h) [61]. The forced expression of ALKBH5 (A5-WT) increases the half-life (3.63 h–7.47 h) of TACC3 in NOMO1 cells compared to mutant A5-Mut. Moreover, TACC3 targets ‘Myc’ and ‘P-21′ genes [92,93], confirmed by decreased Myc and increased P-21 protein levels in ALKBH5-silenced NOMO1 and MMC6 cells [52]. Knockdown of TACC3 showed a similar phenotypic effect like in NOMO1 and MMC6 cells [52]. Collectively, these studies clearly suggest that the m6A-eraser protein ALKBH5 promotes leukemogenesis, and therefore targeting ALKBH5 using selective inhibitors (Selberg et al., 2021, PMID 34056479) could be a potential approach to control AML in humans (Figure 4, Table 2 and Table 3) [52].

### 3.3. Effectors (Readers)

#### 3.3.1. YTHDF2 in AML

**Figure 5:**
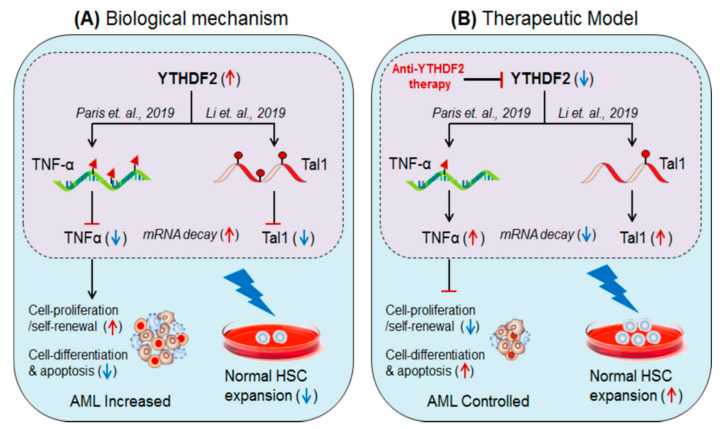
Therapeutic model targeting intracellular m6A-reader protein ‘YTHDF2′ in controlling AML, and in expanding normal HSCs for BM-transplant. (**A**) Biological mechanisms: YTHDF2 facilitates leukaemia progression by suppressing TNF-α [54]. Whereas, hyper-methylation of Tal1 causes HSC-limitations during BM/stem cell transplant [53] (**B**) Therapeutic model: Anti-YTHDF2 therapy controls AML progression as well as allows normal HSCs expansion in vitro by targeting respective genes via mRNA-decay mechanism.

Paris et al., 2019 [54], demonstrated the role of m6A-reader protein ‘YTHDF2’ (YTH N6-Methyladenosine RNA Binding Protein 2) in promoting AML. Authors found that YTHDF2 is required for the development of leukaemia stem cells (LSC) but also initiates AML development [54]. However, selective depletion of YTHDF2 inhibits the self-renewal capacity of leukemic cells and promotes cell-differentiation and apoptosis, suggesting the therapeutic value of YTHDF2 in controlling AML progression. YTHDF2 targets tumour necrosis factor-α (TNF-α) [94], and inhibits its expression by m6A-mediated mRNA decay mechanism [58]. Selective removal of YTHDF2 increases the expression of TNF-α by reducing mRNA decay, resulting in increased terminal cell-differentiation and apoptosis. This result suggests that YHTDF2 plays a crucial role in promoting leukemogenesis [53] (Figure 5).

#### 3.3.2. YTHDF2 in Stem Cell Expansion

Li et al., 2019 [53]; have demonstrated the therapeutic value of m6A-reader protein ‘YTHDF2’ in treating haematological disorders, especially AML including other types of cancers. The hematopoietic stem progenitor cell (HSPC/CD34^+^CD38^−^) population is believed to be a major limiting issue during stem cell transplantation therapy, due to a lesser number of HSPCs populations from a single human umbilical cord blood donor. Therefore, ex-vivo expansion of HSPC population is a major and encouraging challenge for its widespread use. Under steady-state, the YTHDF2 sequesters its target gene T-cell acute lymphocytic leukaemia 1 (Tal1), which is required for the normal proliferation and self-renewal of HSPCs [95,96] and inhibits its function by m6A-marked mRNA-decay mechanism [58]. The selective depletion of YTHDF2 (YTHDF2^KO^) in HSPCs or hUCB-HSCs significantly boosted (10-fold increase) the number of HSPCs by rescuing the expression of Tal1 gene by enhancing mRNA stability. The requirement of YTHDF2 in the maintenance of HSCs, by targeting pro-inflammatory cytokines, was also demonstrated by Mapperley et al., 2020 [97] (Figure 5). These results suggest that YTHDF2 has therapeutic potential and can be clinically used to expand ex-vivo population of normal HSPCs by selective silencing using specific inhibitors of YTHDF2 [53].

## 4. Therapeutic Strategies

### 4.1. Mettl3 Inhibitor

Recently, Eliza et al., 2021 [48] have discovered the selective inhibitor of Mettl3 and Mettl14 (STM2457, IC50 = 16.9 nM) in controlling AML via high-throughput screening of 250,000 drug-like compounds. Functionally, authors showed a significant effect of STM2457 in reducing clonogenic potential and inducing apoptosis in human and mouse AML model without affecting normal human cord blood (CD34^+^/HSPCs) and non-leukaemia (HPC7) hematopoietic cells [48]. Moreover, in vivo studies on AML patient-derived xenograft (PDX) model and primary murine MLL-AF9/Flt3^ltd/+^ model showed convincing anti-leukaemia effect of STM2120 inhibitors [48]. Similar findings have been reported from other research groups summarised in Table 3 [6,45,47].

### 4.2. FTO Inhibitor

Huang et al., 2019 [75], have examined the therapeutic efficacies of two synthetic small molecule inhibitor of FTO (FB23 and FB23-2) in controlling AML. The FTO inhibitor especially ‘FB23-2′ dramatically suppresses cell-proliferation and promotes differentiation and apoptosis of both the cell lines as well as the primary cells in xeno-transplanted mice. Mechanistically, FTO inhibitors directly bind to the FTO and selectively inhibit its m6A-demethylase activity [75]. This result suggests that these FTO inhibitors (FB23 and FB23-2) could be a potential druggable candidate to treat leukaemia [7,19] (Table 3).

### 4.3. FTO in Anti-Tumour Immunity (AML)

**Figure 6:**
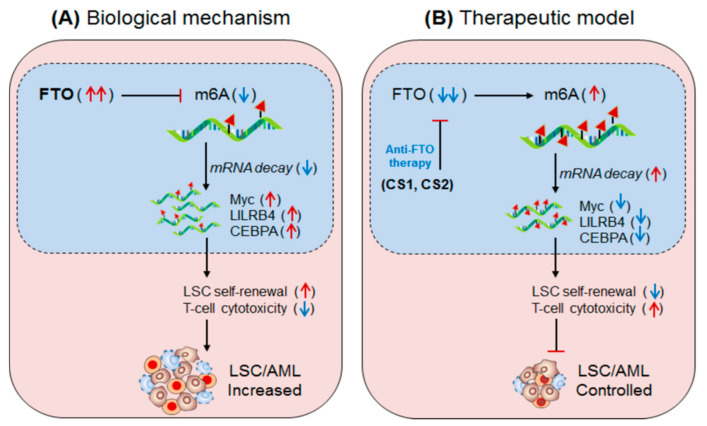
Therapeutic model targeting m6A-eraser ‘FTO’ in controlling LSC progression by enhancing anti-tumour immunity. (**A**) Biological mechanism: FTO is aberrantly expressed in AML, especially LSC populations, and facilitates its progression. Mechanistically, the FTO enhance the expression of its target LILRB4 (immune checkpoint) and other Myc and CEBPA genes via its m6A-demethylase activity by inhibiting m6A-reader protein YTHDF2-mediated mRNA-decay mechanism, causing decreased T-cell activity and increased self-renewal capacity, resulting in enhanced LSC progression. (**B**) Therapeutic model: Anti-FTO therapy: The two synthetic small-molecule inhibitors of FTO (CS1 and CS2) selectively binds to the FTO domain and inhibits its demethylase activity, leading to decreased expression of its target (LILRB4, MYC and CEBPA) mRNA via reducing YTHDF2-mediated mRNA-stability. The decreased expression of the targeted gene ultimately enhances T-cell cytotoxicity and immune evasion and thereby reduced self-renewal capacities, resulting in better control over LSC propagation.

Su et al., 2020 [81] have demonstrated the clinical significance of two synthetic small-molecule inhibitor of FTO, designed on the basis structural guided tool, (CS1 and CS2) in controlling AML, especially leukaemia stem cell (LSC) progression. The abundantly expressed FTO facilitates LSCs progression by impairing T-cell activity via increasing the expression of an immune inhibitory checkpoint molecule/gene ‘LILRB4’. The application CS1 and CS2 significantly controls LSC progression in mouse model with low drug toxicity [81]. Mechanistically, CS1 and CS2 were found to selectively bind to the FTO and inhibit its demethylase activity, resulting in decreased expression of ‘LILRB4’ via inhibiting YTHDF2-mediated mRNA-decay mechanism. Moreover, increased T-cell cytotoxicity and reduced self-renewal abilities of AML cells were noted in the treated mice, suggesting that CS1 and CS2 are potent inhibitors of FTO and can be developed as potential therapeutics against LSC by increasing anti-leukaemia T-cell activity [82]. This hypothesis was further supported by Olsen et al., 2020 [80], (Figure 6). Additionally, some other FTO inhibitors are well-described in these articles [78,98,99,100].

## 5. MicroRNA in AML

### 5.1. miR-150 in AML

In addition to m6A-modifiers, microRNAs also showed promising outcome in the treatment of acute myeloid leukaemia (AML). Fang et al., 2016 [101] have demonstrated the importance of miR-150 in controlling leukaemia progression. Authors showed that miR-150 is downregulated in AML and CML patients, but normalized after complete remission/treatment. However, miR-150 restoration therapy (miR-150 mimic) significantly inhibited the AML by reducing cell-proliferation and promoting apoptosis of the leukaemia stem cells, leading to reduced tumorigenicity in xenograft leukaemia model. The study of underlying mechanism revealed that miR-150 targeted genes are mainly associated with RNA metabolism (synthesis, export, splicing and stability), transcriptional regulation, Wnt-signalling and mammalian target of rapamycin (mTOR)signalling pathways. Interestingly, knockdown of any of these miR-150 downregulated targets (TET3, EIF4B, FOXO4B and PRKCA) showed anti-leukaemia activity similar to miR-150 restoration therapy [102]. Conclusively, these results authenticate the druggable value of miR-150 in treating AML and as a novel candidate for therapeutic drug development [101].

### 5.2. miR-34a in AML

miR-34a was shown to be another microRNA playing a crucial role in controlling elderly AML, who were ineligible for conventional chemotherapy. The authors [103] have found that, the combination of decitabine (dacogen/DAC; known as 5-aza-2′-deoxycytidine) and ATRA effectively control AML and prolong overall survival rate. The combination of DAC and ATRA inhibits the expression of DNA methyltransferase 1 (DNMT1) resulting in activation of miR-34a by hypomethylation. The activated miR-34a further inhibits the expression of Myc [104,105] resulting in cell cycle arrest and increased apoptosis in vitro. These results suggest that miR-34a could be a druggable candidate to control AML progression by modulating miR-34a/Myc axis [103,104,105].

### 5.3. miR-29b in AML

Liu et al., 2019 [106]; demonstrated that miR-29b is another anti-AML candidate microRNA that play an important role in controlling leukaemia stem cell progression, a subtype of acute myeloid leukaemia, characterised by increased self-renewal capacity and decreased apoptosis, by targeting LSC-fucosylation. The authors found that fucosyltransferase 4 (FUT4) is overexpressed in LSC population (CD34^+^CD38^−^) compared to the non-LSC (CD34^+^CD38^+^, CD34^−^CD38^+^, CD34^−^CD38^−^) populations isolated from MOLM13 and KG-1a cell lines determined by LTL lectin assays [106]. However, selective depletion of FUT4 using shRNA significantly decreased cell-proliferation and induced apoptosis of the LSCs. Moreover, in-vitro application of chemotherapeutic drug (ADR, Ara-C and Paclitaxel) in combination with shFUT4-LSC drastically reduced LSC cell-proliferation as compared to shFUT4 alone and drug alone. Furthermore, mouse xenograft studies showed decreased tumour growth when given in combination with shFUT4 and ADR relative to the individual treatments. These results suggest that (i) FUT4-mediated fucosylation facilitate LSCs progression. (ii) FUT4 silencing overcome chemotherapeutic drug resistance in AML.

Mechanistically, authors showed that FUT4 is regulated by specificity protein 1 (Sp1) transcription factor. Interestingly, in silico analysis showed that miR-29b can target Sp1 transcript and inhibits its expression along with Wnt/β-catenin activation. miR29b-mimic inhibits and anti-miR29b promotes Sp1 expression [106] and thus influences FUT4 expression accordingly. Moreover, Wnt/β-catenin pathway inhibition by DKK inhibitor induces apoptosis and reduces cell-proliferation. Taken together, these investigations suggest that miR-29b could be a potential candidate to control LSC progression by regulating miR-29b/Sp1/FUT4 axis [106].

## 6. Suppressor of Cytokine Signalling (SOCS/CISH) in AML

In addition to epigenetic modifiers (RMDs) and microRNAs; the role of cytokine-inducible SH2-domain-containing protein (CISH or CIS) as anti-tumour and anti-leukaemia activity, also, cannot be denied because of its versatile role in regulating cytokine signalling via sensitizing immune cells. For example, (i) CISH knockdown has been shown in increasing anti-tumour immunity by enhancing CD8^+^T-cell effector function [107]. (ii) CISH-knockdown also increases NK-cell fitness and cytotoxicity [108] and thus playing a crucial role in protecting tumour metastasis [109]. (iii) More relevantly, CISH has been shown in maintaining T-cell homeostasis via Mettl3-mediated methylation mechanism [110,111] and (iv) The m6A-reader protein ‘YTHDF2’ is involved in controlling NK cell-mediated anti-tumour immunity [112]. Furthermore, the specific function of CISH in association with acute myeloid leukaemia was demonstrated by Zhu et al., 2020 [113], where the importance of CISH in NK cell-mediated anti-leukaemia activity has been described. They have showed that selective depletion of CISH in NK cells (CISH^−/−^) derived from human iPSC (induced pluripotent stem cells) significantly enhance the expansion as well as survival in the tumour microenvironment, confirmed by improved metabolic fitness of NK-cells, characterised by increased glycolytic capacity and mitochondrial respiration (OxPhos activity) via mTOR signalling response. Moreover, increased NK-cell cytotoxicity was reported against multiple tumour cell lines as well as in in-vivo leukaemia xenograft models [113]. This result additionally supports the role of CISH as an alternative approach to control AML by encompassing immune cell-based therapeutics. Nevertheless, the role of professional antigen-presenting cells like dendritic cells also cannot be circumvented for its proven role in improving anti-tumour immunity by targeting CISH [114,115,116]. One more relevant research outcome showed the involvement of SOCS1, one of the members of CISH/SOCS family, in mimicking AML like phenotype. The overexpression of SOCS1 in zebrafish model showed increased myelopoiesis with distorted kidney and splenic morphology, suggesting the therapeutic inference of AML by targeting SOCS1 (Figure 1) [117].

## 7. Conclusions

In this review, we have described the potential of m6A-modifiers in developing cancer precision medicines and explained how ‘epitranscriptomics’ plays a central role in regulating the crucial genes associated with AML. Among these, the m6A-writer proteins Mettl3 and Mettl14 have been found to be aberrantly expressed in specific subtypes of AML and promotes leukemogenesis by regulating MYB, MYC, Bcl2, pTEN, and PI3K-AKT pathways [43,44,49,118]. Likewise, m6A-methylases (writer), m6A-demethylases FTO, and ALKBH5 (eraser) have also been found to be aberrantly expressed in APL and LSCs/LICs and promote leukemogenesis by targeting ASB2, RARA, LILRB4, CEBPA, TACC3, Myc, and the P-21 gene [51]. Nevertheless, FTO single nucleotide polymorphisms have been also found to be associated with other cancers [119]. Likewise, the reader protein ‘YTHDF2′ has been also found to promote leukemogenesis by inhibiting its targets Tla1 [53] and TNF-α [54] through universal YTHDF2-mediated mRNA decay mechanisms [58]. These discoveries suggest the different mechanism of action of these ‘epigenetics modifiers’ in controlling AML by regulating respective gene targets. However, still some questions remained unanswered as to how all these epigenetic modifiers are upregulated simultaneously and facilitate AML development. The microRNA—miR-150, miR-34a, and miR-29b—have also shown significant anti-leukaemia effects by targeting wnt-signalling, mTOR signalling pathways [101], MYC [103], and Sp1/FUT4-fucosylations [106]. In addition to this, immune-based therapeutics should also be considered in AML treatment by utilizing dendritic cells, T-cells, and NK cell-targeting CISH-mediated signalling [110,112,115]. Therefore, it is very critical to understand the stages of AML progression to implement these exciting RMDs in eradicating AML with other forms of standard chemotherapeutic drugs [120].

## 8. Future Prospective

Targeting m6A-modification machineries has revolutionised the class of epigenetic research in discovering novel therapeutic drug targets. Co-targeting of intracellular genes along with m6A-modification machineries will improve the level of immunotherapy against those cancers that are resistant to the immune checkpoint-based therapeutics in patients with blood cancer. Not far in the future targeted therapy in combination with other drugs may become a universal panacea to control many diseases and other forms of cancer. Moreover, targeting suppressors of the cytokine signalling family of proteins would further potentiate the immunotherapeutic potential of RNA modification inhibitors by targeting epigenetic regulator of dendritic cells. Pharmaceutical approaches pertaining to RNA epigenetic modification machinery are expected to shed light on the field of cancer immunotherapy for AML and other forms of blood cancer. Nevertheless, several human clinical trials are required to access the therapeutic value of these inhibitors.

Box 1 **Key definitions.**

**Epitranscriptomics**: refers to the post transcription RNA modifications that regulate the activity of the RNA molecule without affecting RNA sequence. The major epitranscriptomic machineries are Writer (editor), Eraser (remover) and Reader (effector) that govern this emerging [121].

**Writers**: are the main epitranscriptomic enzymes that incorporate methyl group to various residues of the transcript. Some examples of writers are Mettl-3, Mettl-14, Mettl-16, WTAP, KIAA1429, RBM15/RBM15B and ZC3H13.

**Erasers**: are the main epitranscriptomic enzymes that remove methyl group added by the writers. Some examples of erasers are FTO, ALKBH-3 and ALKBH-5.

**Readers**: are the main epitranscriptomic enzymes/proteins that read n6A-marked transcripts and affect its regulation. Some examples of Reader are YTHDF-1, YTHDF-2, YTHDF-3, YTHDC-1, YTHDC-2 and ELAV1.

## Figures and Tables

**Table 1:** Therapeutic drug for acute myeloid leukaemia.

Conventional Drugs				
Brand/Other Name	Drug	Drug Type	Clinical Trial	Ref.
Rydapt (Novartis)	Midostaurin	Multikinase FLT3 inhibitor	FDA approved	[29]
Vyxeos (Jazz Pharma)	CPX-351	cytarabine and daunorubicin combination (5:1 molar ratio)	FDA approved	[30,31]
Formerly SNS-595 (Sunesis Pharma)	Vosaroxin	Topoisomerase II inhibitor: anticancer quinolone derivative (AQD)	FDA approved	[32]
ASP2215, Xospata (Astellas Pharma)	Gilteritinib	Dual inhibitor of FLT3/AXL	FDA approved	[33,34]
Venclexta, Venclyxto (AbbVie, Genentech)	Venetoclax	Bcl2-inhibitor	Phase III	NCT02993523 NCT03069352 [35]
**Epigenetic Drugs**				
Vidaza	Azacitidine	DNMT inhibitor (Hypomethylating agent)	FDA approved	[19]
Dacogen	Decitabine	DNMT inhibitor (Hypomethylating agent)	FDA approved	[19]
Tibsovo (AG-120)	Ivosidenib	IDH1 inhibitor	FDA approved	[23,24]
Idhifa (AG-221)	Enasidenib	IDH2 inhibitor	FDA approved	
Beleodaq (PXD101)	Belinostat	Pan-HDAC inhibitor	Phase II	[36]
Zolin	Vorinostat	Pan-HDAC inhibitor	Phase I/II	[37]
–	Panobinostat	Pan-HDAC inhibitor	Phase I/II	[38,39,40]
Istodax	Romidepsin	Selective HDAC inhibitor	Preclinical	[28]
SGI-110	Guadecitabine	Dinucleotide of decitabine and deoxyguanosine	Phase III	NCT02348489
**Monoclonal Antibodies (mAbs)**				
GO (Mylotarg, Wyeth Pharma)	Gemtuzumab Ozogamicin	CD33-targeted	Phase 2	NCT03374332 NCT00372593
SGN-CD33A (Seattle Genetics)	Vadastuximab talirine	CD33-targeted	Phase I	[41]
Darzalex Faspro (Janssen Biotech)	Daratumumab	CD38-targeted	Phase II	NCT03067571
	RG7356	CD44-targeted	Phase I	[42]
Iomab-B	Apamistamab	CD45-targeted	Phase III, SIERRA	NCT02665065

**Table 2:** Players of RNA epigenetic machineries as therapeutic targets in AML.

RNA Modifiers	Disease Condition	Target	Mechanism of Action	Therapeutic Strategies	Ref.
Writers Mettl3	upregulated in AML	MYB/MYC, Bcl2, pTEN, Spl1 (PU.1), PI3K-AKT pathway	Inhibiting cell-differentiation and apoptosis and promoting cell proliferation (self-renewal) capacity.	Selective Mettl-3/14 inhibitor or Targeted therapy	[43,46]
Mettl-14					[46,47,48,49]
Erasers FTO	upregulated in APL and LSC/LICs	Myc, CEBPA	Regulated by FTO related with Leukaemia		[50]
		ASB2, RARA, MYC, LILRB4, CEBPA	By inhibiting ASB2 and RARA gene targets as well as ATRA-induced cell-differentiation and apoptosis	Targeted silencing or specific FTO/ALKBH5 inhibitor	[51]
ALKBH5		TACC3, Myc, P21	By impairing self-renewal capacities		[52]
Readers YTHDF2	upregulated in AML	Tal1	YTHDF2 inhibit the expression of essential *Tal1* gene.	Targeted silencing/ therapy	[53]
YTHDF2	upregulated	TNFα	YTHDF2 inhibit the expression of TNFα required for cell necrosis and apoptosis.		[54]

**Table 3:** Selective inhibitors of m6A-modifiers in AML treatment.

Name		Therapeutic Application		Ref.
	Disease	Epigenetic Inhibitor	Function	
Writers Mettl3, Mettl14 Inhibitors	AML	STM2457 (STC-15)	By selective inhibition of Mettl-3/14 (proposed human clinical trial in 2022 by Storm Therapeutics)	[45,47,48]
	Metabolic disease	SAH (S-Adenosyl-homo cysteine)	Selective inhibition of Mettl-3/14 by targeting endogenous metabolite	[76]
	Cancer	UZH1/UZH1a	Promotes apoptosis and enhance T-cell anti-tumour activity	[45,77]
Erasers FTO Inhibitors	Upregulated in AML	FB23	Selective FTO inhibitor works by retaining m6A-demethylase activity in LSCs	[75,78]
		FB23-2		[7,75]
		R-2-hydroxyglutarate (R-2HG)	Selective FTO inhibitor targeting A/MYC/CEBPα signalling and increase anti-tumour immunity	[79]
		CS1 and CS2	Selective FTO inhibitor targeting LILRB4 immune checkpoint gene	[80,81]
	Glioblastoma Stem Cells	Non-steroidal anti-inflammatory drug, (MA2) an ethyl ester form of meclofenamic acid	Selective FTO inhibitor targeting ADAM19 gene by m6A hyper-methylation	[82,83]
	Triple-negative inflammatory breast cancer	MO-I-500	Selective inhibitor of FTO targeting IRX3 gene in SUM149-MA cells	[84]
	CNS (epilepsy)	BBB-penetrating small molecule inhibitor of FTO	First FTO inhibitor with anticonvulsant activity by targeting various microRNAs	[85]
Readers YTHDF2 Inhibitors	AML	BET inhibitor (OTX015)	Bromodomain inhibition	[86,87,88]
		MB-3 inhibitor of KAT2A	MB-3 inhibits the expression of KAT2A gene	[89,90]

## Data Availability

Not applicable.
